# POLYPOID BASAL CELL CARCINOMA MASQUERADING AS PYOGENIC GRANULOMA

**DOI:** 10.4103/0019-5154.70681

**Published:** 2010

**Authors:** S Yadav, G P Thami, A Bhatnagar, S Gill

**Affiliations:** *From the Department of Dermatology and Histopathology, Government Medical College and Hospital, Chandigarh, India. E-mail: savita2081@gmail.com*

Sir,

Basal cell carcinoma (BCC) is a malignant tumor composed of basaloid cells that arise from basal cells of the epidermis or epithelial structures of the adnexa. Clinically and histologically it may have varying appearances. Rarely, BCC presents as a polypoidal growth.

Polypoid BCC differs clinically from other variants by being exophytic and pedunculated and, histologically, the tumor aggregation is restricted only to the polypoid part. Although the polypoid BCCs have a large average size, they are not considered aggressive because the lesions are well-circumscribed and the growth pattern is non-infiltrative.

A 57-year-old lady with skin type V presented with a continuously growing mass on the left cheek for two years. The lesion used to bleed with minimal trauma. The tumor was located on the left nasolabial fold, polypoidal in shape, bluish black in color, soft to firm in consistency and measured approximately 3 × 2 cm in size [Figure 1]. There was no associated cervical lymphadenopathy and systemic examination was unremarkable. The differential diagnoses considered were BCC, pyogenic granuloma and adnexal tumor. The tumor was excised with 5 mm margins, followed by two freeze thaw cycles of cryotherapy at the base. There was no recurrence at six months of follow-up.

**Figure 1:**
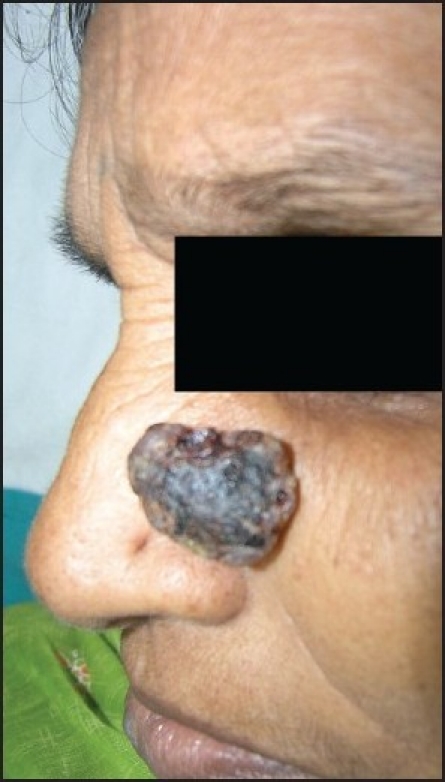
Polypoid BCC on the left nasolabial fold of the lady

The histological examination of the mass revealed tumor cells arising from the basal layer of the overlying thinned and stretched out epidermis [Figure 2]. The tumor cells were arranged in the form of solid sheets, nests and areas showing adenoid pattern. Tumor cells were basaloid with uniform nuclei and scanty amount of cytoplasm. Peripheral palisading of the tumor cells were seen with areas showing retraction artifact. Few mitotic figures and areas of keratinization were noted. The margins of the excised tissue were tumor-free.

**Figure 2:**
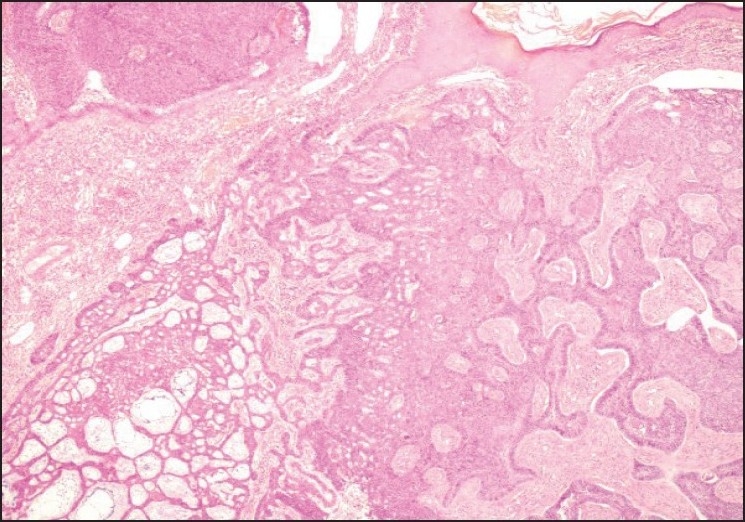
The figure shows peripheral palisading of basal cells with characteristic retraction artifacts along with focal gland formation (H and E stain, ×100)

There are different clinical and histologic variants of BCC. The clinical variants are ulcerating, nodular, pigmented, sclerodermiform and superficial types. The histological variants are nodular, noduloulcerative, micronodular, pigmented, fibroepithelial, adenoid, adenoid cystic, morphoeaform, bowenoid, basosquamous, infiltrative, superficial, adamantinoid, keloidal, syringomatous, cylindromatous, calcifying, ossifying and BCC with follicular, eccrine, apocrine and sebaceous differentiation.

Polypoidal BCC is a rare presentation which was first reported in 1985 by Love *et al*.[1] It has a stalk that connects it to the skin surface. Histologically, it has pedunculated exophytic growth and is characterized by restriction of the tumor aggregations to the polypoid zone. In the largest reported case series of polypoidal BCC by Megahed[2] there were four cases and, in one, the BCC appeared over naevus sebaceous.

Polypoidal BCC has been described more commonly in females as in our case. Misago *et al*.[3] reviewed the literature and found that the peculiar favorable locations of polypoidal BCC are scalp, genital area, back and buttocks. However, in our case the lesion was present on the face, which has been uncommonly described. Another unusual feature was the history of recurrent bleeding with minimal trauma and the bluish black appearance of the tumor resembling pyogenic granuloma.

Hence our case represents a unique polypoidal BCC on the face masquerading as pyogenic granuloma. The polypoid BCC reported here is a rare clinical variant and to the best of our knowledge, has never been reported from India.

## References

[1] Love GL, Sarma DP (1985). Giant polypoid basal cell carcinoma. J Surg Oncoll.

[2] Megahed M (1999). Polypoid basal cell carcinoma: a new clinicopathological variant. Br J Dermatol.

[3] Misago N, Narisawa Y (2004). Polypoid Basal cell carcinoma on the perianal region: a case report and review of the literature. J Dermatol.

